# The predictive value of GLIM criteria on clinical outcomes and responses to nutritional support in patients with neurocritical illnesses

**DOI:** 10.1038/s41598-024-65994-2

**Published:** 2024-07-01

**Authors:** Peiqi Liu, Huimin Tian, Lan Gao, Tangsheng Zhong, Yujiao Wang, Li Chen

**Affiliations:** 1 School of Nursing, Jilin University, No. 965 Xinjiang Street, Chang Chun, 130021 Jilin Province China; 2 Department of Neurology, The First Hospital of Jilin University, No. 71 Xinmin Street, Changchun, 130021 Jilin Province China; 3 Neurocritical Care Unit, Department of Neurology, The First Hospital of Jilin University, No. 71 Xinmin Street, Changchun, 130021 Jilin Province China; 4 Department of Pharmacology, College of Basic Medical Sciences, Jilin University, Changchun, 130021 Jilin Province China

**Keywords:** GLIM, Malnutrition, Nutritional assessment, Neurocritical ill, Clinical outcome, Nutrition, Neurological disorders

## Abstract

Neurocritically ill patients frequently exhibit coma, gastroparesis, and intense catabolism, leading to an increased risk of malnutrition. The Global Leadership Initiative on Malnutrition (GLIM) criteria for the diagnosis of malnutrition was created to achieve a consistent malnutrition diagnosis across diverse populations. This study aimed to validate the concurrent and predictive validity of GLIM criteria in patients with neurocritical illnesses. A total of 135 participants were followed from admission to the neurocritical unit (NCU) until discharge. Comparing GLIM criteria to the Subjective Global Assessment (SGA), sensitivity was 0.95 and specificity was 0.69. Predictive validity of GLIM criteria was assessed using a composite adverse clinical outcome, comprising mortality and various major complications. Adjusted hazard ratios for moderate and severe malnutrition were 2.86 (95% CI 1.45–5.67) and 3.88 (95% CI 1.51–9.94), respectively. Changes in indicators of nutritional status, including skeletal muscle mass and abdominal fat mass, within 7 days of admission were obtained for 61 participants to validate the predictive capability of the GLIM criteria for the patients’ response of standardized nutritional support. The GLIM criteria have a statistically significant predictive validity on changes in rectus femoris muscle thickness and midarm muscle circumference. In conclusion, the GLIM criteria demonstrate high sensitivity for diagnosing malnutrition in neurocritically ill patients and exhibit good predictive validity.

## Introduction

Malnutrition is widely recognized as a condition related to an increased overall risk and higher disease burden worldwide^1^. Approximately 20%–50% of hospitalised patients suffer from malnutrition^2,3^, and a worsening of nutritional status leads to a longer length of stay in hospital, impaired functional status, higher rate of complications, and even death^3,4^. Despite its significant impact, the identification of malnutrition lacked global consensus^5^ until the publication of diagnostic criteria by the Global Leadership Initiative on Malnutrition (GLIM) working group in 2018. This criterion is based on widely used items; however, as a global consensus, it needs to be verified in various regions and populations worldwide.

Neurocritical care represents a well-established subspecialty offering specialized treatment and care to critically ill patients with neurological diseases or neurological manifestations of systemic diseases^6^. Compared to the ICUs, patients treated in the neurocritical care units (NCUs) tend to have a longer length of critical care stays and lower mortality, as well as a higher demand for nutritional support^7^. Moreover, patients with neurocritical illness frequently encounter coma, gastroparesis, intense catabolism, movement restrictions, and dysphagia, all of which can significantly affect their nutritional status^8,9^, leading to a serious challenge in identifying malnutrition in the NCU. Timely identification of malnutrition via nutritional assessment is essential for providing personalized nutritional support, predicting, and improving patient outcomes^10^. Currently, there is no gold standard for diagnosing malnutrition in critically ill patients^11^, and few studies have validated the GLIM criteria in neurocritically ill patients.

Validation of the predictive validity of the GLIM criteria on health outcomes, including in-hospital mortality and major complications, is recommended by the GLIM working group^12^ and is considered an indirect form of supplementary validation^13^. Several studies have verified the predictive validity of GLIM criteria on adverse clinical outcomes in ICU patients^14^, including mechanical ventilation, length of ICU stay, and mortality^15,16^. In addition, the changes of muscle mass during hospital stay could reflect the validity of nutritional support, and it is holding significant importance in guiding clinical nutrition practice. However, studies validating the predictive value of the GLIM criteria on muscle mass changes in neurocritically ill patients are currently lacking.

To address these knowledge gaps, we conducted a prospective observational study following the recommendations of the GLIM consensus to verify the predictive ability of the GLIM criteria for adverse clinical outcomes and muscle mass changes in patients with neurocritical illness. Additionally, we verified the concurrence of the GLIM criteria for malnutrition using the semi-gold standard Subjective Global Assessment (SGA). The aim of our study is to facilitate the adoption of GLIM criteria for diagnosing malnutrition in patients with neurocritical illnesses.

## Materials and methods

### Study design and participants

Single-center, prospective observational study carried in a 24 beds NCU of an academic center. Patients older than 18 years, admitted to the NCU and expected to stay for more than 48 h were included in the study. Informed consent for participation in the study was obtained from the patients’ families. Patients who refused treatment, sign do-not-resuscitate (DNR) orders, had a second admission within 6 months, were unable to complete the nutritional assessment, were pregnant, or had a mental illness were excluded. Participants were followed from admission to NCU until discharge. This study was approved by the Ethics Committee of the School of Nursing, Jilin University (No. 2023040602), and conducted by an independent team outside clinical care, was not involved in any clinical decisions or interventions. This study performed in accordance with the Declaration of Helsinki and registered with the Chinese Clinical Trial Registry Before Recruitment (ChiCTR2300070714). The report of this study followed the Strengthening the Reporting of Observational Studies in Epidemiology—Nutritional Epidemiology (STROBE-nut) guideline.

### Clinical characteristics and measurements

Characteristics and medical information including age, sex, height, weight, date of hospital admission, time of NCU admission, medical history, clinical diagnosis, medical order records, disease course records, and discharge information, were obtained from electronic medical records. The worst Acute Physiology and Chronic Health Evaluation II (APACHE II) score^17^, Sequential Organ Failure Assessment (SOFA) score^18^, Charlson Comorbidity Index (CCI)^19^, Glasgow Coma Scale (GCS)^20^ were obtained within 24 h of admission and used to reflect disease severity at admission.

### Nutritional assessment

#### Ultrasound imaging measurements

Ultrasound imaging was performed within 48 h of admission and at 7 days of NCU stay by a research nurse trained in critical care ultrasound imaging techniques to measure the muscle and fat thickness of participants. A SonoSite M-Turbo (FUJIFILM Sonosite, Inc., Bothell, Washington, USA) ultrasound with a 13-6 MHz linear transducer (type: HFL38x) was used to measure the following parameters: rectus femoris muscle thickness (RFMT), preperitoneal visceral fat thickness in the abdomen (PVFT), and subcutaneous fat thickness in the abdomen (SFT). During ultrasound imaging, the patient was placed in the supine position, the knees relaxed, the legs straightened, and the toes pointed upward. The transducer was placed perpendicular to the skin and femur at the midpoint between the anterior superior iliac spine and the upper lateral epicondyle of the femur^21^ to measure RFMT. The right side was preferred for the measurement; if it could not be approached, the left side was measured. The transducer was placed perpendicular to the skin and parallel to the linea alba and scanned along it from the xiphoid process to the umbilical cord, and the minimum SFT and PVFT were measured at the end of exhalation^22^. All measurements were performed while the patient was relaxed, the pressure of the transducer on the skin was reduced as much as possible, and the site was marked at the first measurement to ensure the consistency of the position before and after. Each indicator was measured three times at the same time point, and the average value was calculated. Ultrasound images were saved on a computer and measured using the ImageJ2 software (National Institutes of Health, Bethesda, Maryland, USA).

#### Anthropometry measurements

Anthropometric measurements were taken by the researchers when the participants were relaxed in a supine position within 48 h of admission and at 7 days of NCU stay. Triceps fold thickness (TFS) was measured at the midpoint of the acromion and olecranon processes of the nondominant arm using a skinfold calliper (Huayi Automation Instrument Co., Ltd., Changshu, Jiangsu, China) with an accuracy of 0.5 mm. The midarm circumference (MAC) was measured at the same point using a measuring tape with an accuracy of 1 mm. The midarm muscle circumference (MAMC) was calculated using the formula reported by Frisancho^23^. Calf circumference (CC) was measured at the thickest part of the calf using the same measuring tape.

#### Malnutrition diagnosis

Malnutrition Diagnosis were performed within 48 h after NCU admission by well-trained research nurses and dietitians. A structured interview with each participant’s families was conducted to gather information for nutritional assessment. The participants' dietary changes, gastrointestinal symptoms, and weight changes were systematically documented post-interview. Nutrition-related data were collected by research nurse and further evaluation by registered dietitians for a diagnosis of malnutrition by using SGA^24^ and GLIM criteria^25^ respectively. The GLIM criteria for diagnosing malnutrition include three phenotypic criteria: weight loss, low Body Mass Index (BMI), and reduced muscle mass, and two etiologic criteria: reduced food intake or assimilation and chronic and/or acute inflammation. Malnutrition was diagnosed when at least one phenotypic and etiological criterion was met. Phenotypic criteria were used to grade malnutrition (stage 1, moderate malnutrition; stage 2, severe malnutrition). According to the assessment of the muscle mass phenotypic criterion for the GLIM diagnosis of malnutrition^26^, ultrasound imaging measurement of the RFMT was used to assess the reduction in muscle mass, < 10 mm in females and < 11 mm in males, according to a study based on Asian populations^27^. Owing to the lack of reference values, RFMT is not applicable for grading malnutrition. The low BMI index uses the standard of Asian people^28^, which is moderate malnutrition: < 18.5 kg/m^2^ for age < 70, < 20 kg/m^2^ for age ≥ 70 and severe malnutrition < 17 kg/m^2^ for age < 70, < 17.8 kg/m^2^ for age > 70. Weight loss > 5% within 6 months or > 10% before 6 months was the criterion for moderate malnutrition, and > 10% within 6 months or > 20% before 6 months was the criterion for severe malnutrition. Reduced food intake or assimilation was obtained from medical or nutritional assessment records; intake reduction > 50% for more than one week, any reduction for more than two weeks, or gastrointestinal symptoms for more than two weeks were positive criteria. Participants with serum C-reactive protein (CRP) > 0.5 mg/dL were considered to be in an inflammatory status.

### Outcome measurements

#### Primary outcome

The primary outcome of our study was adverse clinical outcomes. It is a composite outcome with the following items: 1) clinical death, 2) haemodynamic instability, 3) cardiovascular and cerebrovascular events, 4) gastrointestinal events, 5) blood transfusion, and 6) multidrug-resistant infection. Clinical death was defined as cardiorespiratory arrest declared as death by a physician. Haemodynamic instability was defined as hypotension due to various causes requiring the administration of dopamine or epinephrine. Cardiovascular and cerebrovascular events were defined as new myocardial infarction, cerebral infarction, cerebral haemorrhage, or exacerbation of an existing infarction. Gastrointestinal events were defined as positive faecal occult blood with black or red bloody stool. Transfusion therapy involves the transfusion of whole blood or blood components. Multidrug-resistant infections were defined as positive body fluid cultures for multidrug-resistant bacteria. The occurrence of any of these factors was considered an adverse clinical outcome.

#### Exploratory outcome

Reassessment was performed for participants whose NCU stay reached 7 days to obtain the longitudinal change in nutritional status in participants with or without malnutrition. RFMT, CC, MAMC, PVFT, and SFT were performed within 48 h after 7 days by the same evaluation described above. The daily calorie intake of each patient was calculated based on the intake of enteral and parenteral nutrition substances per day, and recorded in the intensive care records by their assigned nurses. The research nurses obtained the participants' calorie intake by consulting these records to compare the difference of calorie intake between participants with or without malnutrition.

### Sample size

The sample size was estimated using PASS (v15.0; NCSS, LCC, Kaysville, Utah, USA). Based on a previous study^29^, the prevalence of exposure was 0.28, the incidence of outcome in the exposed group was set to 0.42, in the control group was 0.23, and the average time of outcome occurrence was 3 days. Select the two-sided log-rank test, set the expected accrual time to 150 days, the total time to 180 days, type I error (*α*) = 0.05 and statistical power (1-*β*) = 0.8. The estimated effective sample size was 120.

### Statistical methods

A normality test was performed on all continuous variables, and the samples conforming to the normal distribution were described as mean and standard deviation (SD), while the samples that did not conform to the normal distribution were described as median and interquartile range (IQR). For the comparison of differences between groups, the Student’s *t*-test was used between two independent samples that conformed to the normal distribution, and the Mann–Whitney *U* test was used for the samples that did not conform to the normal distribution. Counting variables were analysed using the chi-squared (*Xi*^2^) test. Sensitivity, specificity, Positive Predictive Value (PPV), Negative Predictive Value (NPV); Positive Likelihood Ratio (PLR); and Negative Likelihood Ratio (NLR) were calculated to quantify the diagnostic effect (< 0.5 Poor, 0.5–0.8 Fair, > 0.8 Good). Cohen's *Kappa* coefficient was calculated to quantify consistency with the semi-gold standard (*Kappa* = 0 poor; 0.01–0.2 slight; 0.21–0.4 Fair; 0.41–0.6 Moderate; 0.61–0.8 Substantial; 0.81–1.0 Almost perfect). A Kaplan–Meier curve was used to visually compare the occurrence of the primary outcome in participants with different nutritional statuses, and the log-rank test was used for statistical testing. Proportional hazard regression models were used to calculate the hazard ratios (HRs) of different nutritional statuses and were adjusted for potential confounding factors. Caloric intake in participants with different nutritional statuses was compared using a general linear model, and the interaction effect of group and time on nutritional status was analysed using generalised estimation equations. All statistical tests were performed using SPSS v26.0 (IBM Corp. Armonk, New York, USA) A *P*-value of < 0.05 was considered significant.

### Ethics approval and consent to participate

This study was approved by the Ethics Committee of the School of Nursing, Jilin University (No. 2023040602). Consents were obtained from participants or their legal agent.

## Results

### Participants characteristics

**Figure 1:**
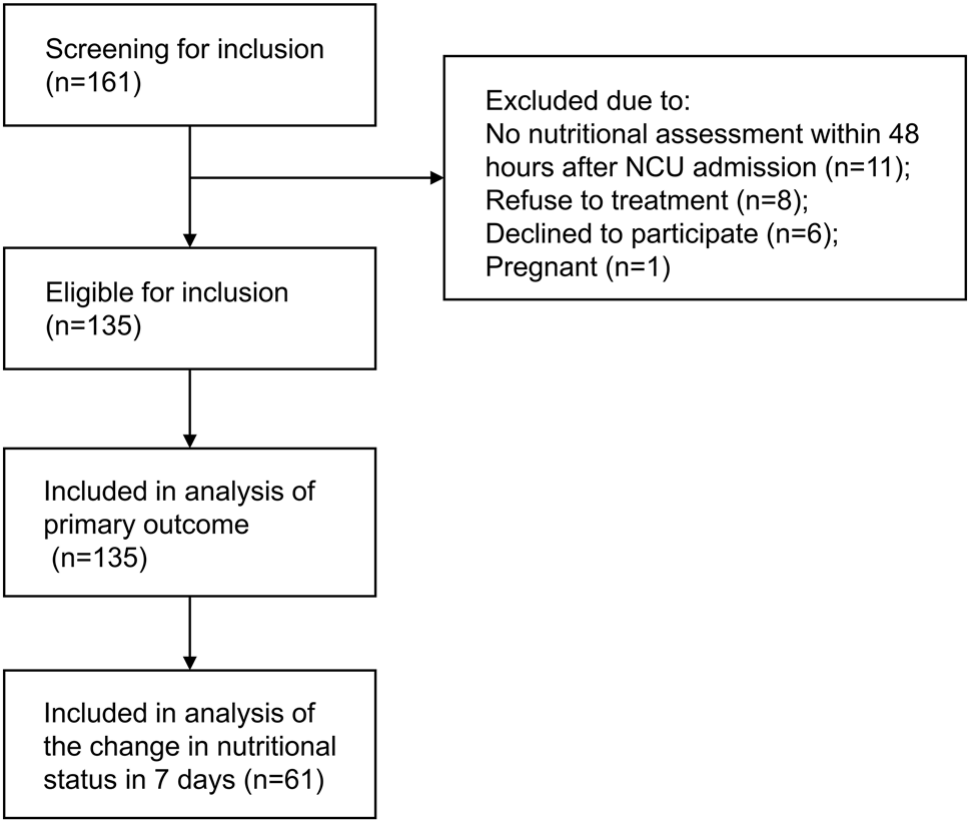
Flow diagram of participants' enrolment.

A total of 161 patients met the inclusion criteria between January and August 2023, after excluding 26 of them. A total of 135 participants were included. Most participants had a primary diagnosis of stroke (n = 101, 74.8%). The Average APACHE II score was 14.36 ± 4.93, and the median GCS score was 9 [7,21]. The median NCU length of stay was 8 [5,15] days. At day 7, 61 (45.2%) participants reassessed their nutritional status in the hospital by the same dietitian and were included in further analysis of the change in nutritional status during the NCU stay (Fig. 1) to reflect the response to nutritional support. The details of the baseline characteristics the participants are shown in Table 1 and Supplementary Material, Table S1.

**Table 1:** Comparison between participants with or without malnutrition defined by GLIM criteria.

	Well-nourished n = 69	Malnourish n = 66	*P*
Age (years)	59.33 ± 12.71	64.98 ± 13.4	0.013
Female	18 (26.1)	32 (48.5)	0.007
Primary diagnosis			
Stroke	50 (72.5)	51 (77.3)	0.421
Encephalitis	10 (14.5)	6 (9.1)	0.332
Epilepsy	5 (7.2)	5 (7.6)	0.942
Myasthenia	4 (5.8)	4 (6.1)	0.948
SOFA	5.11 ± 1.97	5.28 ± 2.15	0.629
APACHE II	14.27 ± 4.68	14.45 ± 5.21	0.834
GCS	9 [6,12]	9 [6,12]	0.523
LOS before NCU admission (days)	0 [0, 1]	0 [0, 1]	0.888
Length of NCU stay (days)	9 [5.5, 15.5]	8 [4, 14.25]	0.695
Antibiotics free days	1 [0, 3]	0 [0, 3]	0.968
Mechanical ventilation	24 (34.8)	21 (31.8)	0.715
NCU transfer out destination			
Home	24 (34.8)	8 (12.1)	0.002
Hospital ward	41 (59.4)	50 (75.8)	0.043
Others^a^	4 (5.8)	8 (12.1)	0.197
Adverse clinical outcome	14 (20.3)	36 (54.5)	< 0.001
Clinical death	3 (4.3)	6 (9.1)	0.269
Hemodynamic instability	7 (10.1)	22 (33.3)	0.001
Cardiovascular events	1 (1.4)	3 (4.5)	0.289
Gastrointestinal events	4 (5.8)	5 (7.6)	0.679
Blood transfusion	3 (4.3)	5 (7.6)	0.427
Multidrug resistant infection	3 (4.3)	6 (9.1)	0.269
BMI (kg/m^2^)	25.86 ± 4.61	22.73 ± 4.48	< 0.001
SGA malnutrition	2 (2.9)	36 (54.5)	< 0.001
RFMT (mm)	14.62 ± 3.52	9.93 ± 3.21	< 0.001
CC (cm)	33.66 ± 3.75	31.29 ± 3.79	< 0.001
MAMC	24.18 ± 4.80	22.84 ± 2.84	0.051
PVFT (mm)	12.69 ± 5.52	9.88 ± 5.23	0.003
SFT (mm),	12.54 ± 5.94	12.22 ± 5.87	0.756
ALB (g/L)	37.99 ± 5.77	36.07 ± 6.28	0.074
Nutritional assessment in day 7	35 (50.7)	26 (39.4)	0.186

*SD* Standard deviation, *SOFA* Sequential Organ Failure Assessment, *APACHE II* Acute Physiology and Chronic Health Evaluation II; Chronic Health Evaluation II; *CCI* Charlson Comorbidity Index, *GCS* Glasgow Coma Scale, *IQR* interquartile range, *LOS* Length of stay, *NCU* Neurocritical Care Unite, *BMI* Body Mass Index, *SGA* Subjective Global Assessment, *RFMT* Rectus femoris muscle thickness, *CC* Calf circumference, *MAMC* Mid-arm muscle circumference, *PVFT* Preperitoneal visceral fat thickness, *SFT* subcutaneous fat thickness, *ALB* Albumin.

^a^Including Long-term care facilities, day care canters, and other nursing facilities, or transfer out of NCU due to death.

### Diagnosis of malnutrition

The prevalence of malnutrition in the participants at NCU admission diagnosed by the SGA and GLIM was 28% and 49%, respectively. Half of the participants were diagnosed with malnutrition, 27% were diagnosed using both criteria, 22% met the GLIM criteria, and only 1% were diagnosed using the SGA criteria. Nearly all the participants with the etiological criteria. The positive rates of each criterion was shown in supplementary material Table S2, the treatment of SGA as a semi-gold standard showed that the GLIM criteria had very good sensitivity (0.95), fair specificity (0.69), and moderate agreement with SGA (*kappa* = 0.52). Considering the diagnostic effect of sub-items in the GLIM criteria, weight loss had substantial consistency with SGA (*kappa* = 0.68) in the phenotypic criteria.

### The predictive value of GLIM criteria on adverse clinical outcome

**Figure 2:**
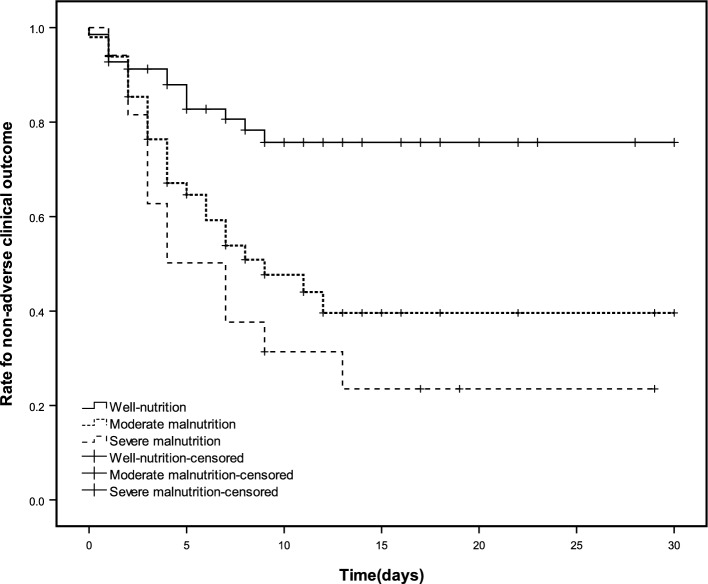
Kaplan–Meier survival curve of the main outcome among different nutritional status.

As shown in Fig. 2. The result of the Kaplan–Meier survival curve suggested that moderate and severe malnutrition determined by the GLIM criteria had a predictive value for adverse clinical outcomes, and the difference in the incidence of adverse clinical outcomes among the well-nourished, moderate malnutrition, and severe malnutrition was proved by the log-rank test (*P* < 0.001). The results of proportional hazards regression models are shown in Table 2, both moderate and severe malnutrition had an capability for predicting adverse clinical outcomes (HR = 2.79, 95% CI 1.44–5.39; HR = 4.10, 95% CI 1.89–8.87, respectively). After adjusting for age, sex, CCI, SOFA, and primary diagnosis, the predictive value of GLIM criteria on adverse clinical outcome maintained significance significant (HR = 2.86, 95% CI 1.45–5.67; HR = 3.88, 95% CI 1.51–9.94, respectively). In subentries, both moderate and severe malnutrition had the predictive value of haemodynamic instability after adjusting (HR = 2.62, 95% CI 1.02–6.70; HR = 3.67, 95% CI 1.13–11.96, respectively). The predictive value of severe malnutrition on clinical death was significant in the crude model (HR = 4.10, 95% CI 1.34–26.90), but not in the adjusted model (HR = 3.15, 95% CI 0.39–25.42).

**Table 2:** Results of proportional hazards regression models for GLIM criteria malnutrition and adverse clinical outcomes (HRs with 95% CI).

Outcome	Moderate malnourished(n = 49)^a^		Severe malnourished(n = 17)^a^	
n = 135	Crude	Adjusted^b^	Crude	Adjusted^b^
Adverse clinical outcome	2.79 (1.44–5.39)	2.86 (1.45–5.67)	4.10 (1.89–8.87)	3.88 (1.51–9.94)
Clinical death	0.94 (0.16–5.61)	0.56 (0.85–3.77)	6.01 (1.34–26.90)	3.15 (0.39–25.42)
Hemodynamic instability	3.08 (1.24–7.63)	2.62 (1.02–6.70)	5.35 (1.94–14.78)	3.67 (1.13–11.96)
Cardiovascular events	2.87 (0.26–31.66)	3.50 (0.29–41.72)	4.53 (0.28–72.39)	10.51 (0.29–378.43)
Gastrointestinal events	1.51 (0.38–6.06)	1.89 (0.44–8.08)	1.06 (0.12–9.50)	2.20 (0.17–28.89)
Blood transfusion	2.46 (0.59–10.31)	5.58 (0.87–35.73)	–	–
Multidrug resistant infection	2.43 (0.58–10.18)	3.29 (0.72–14.99)	1.69 (0.18–16.32)	1.74 (0.12–24.50)

*HR* hazard ratio, *CI* confidence interval, *SOFA* Sequential Organ Failure Assessment, *CCI* Charlson Comorbidity Index.

^a^Reference to well-nutrition participants. HRs was calculated by using proportional hazards regression models.

^b^Adjusted for age, sex, primary diagnosis, SOFA score, and CCI score.

### The predictive value of GLIM criteria on the effect of nutritional support

**Figure 3:**
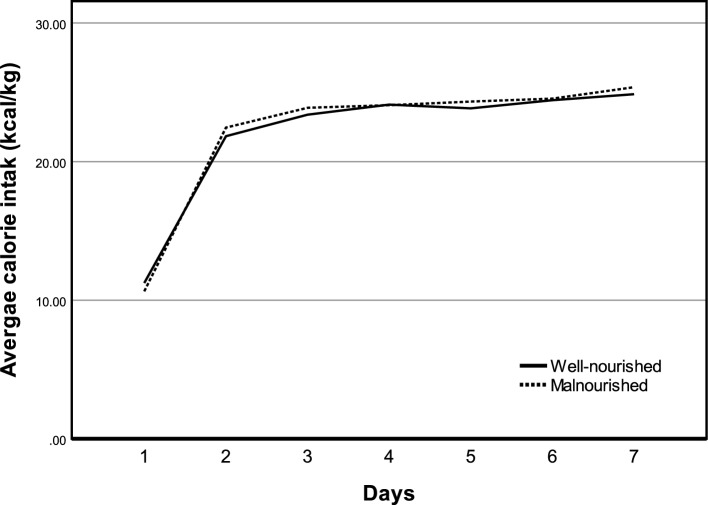
Average calorie intake between patients with or without GLIM defined malnutrition in the first 7 days (*F* = 0.023, *P* = 0.881).

The general line model was used to analyse the differences in nutritional delivery between participants with and without malnutrition. As shown in Fig. 3 and Supplementary Material Table S3, the differences between participants with and without malnutrition were not statistically significant (*F* = 0.023, *P* = 0.881). Reassessment of their nutritional status was given to 61 participants on days 7 and 35 in the well-nourished group and to 26 in the malnourished group, and no statistical difference was found between the two groups (Table 1, *P* = 0.186). A generalised estimating equation was used to evaluate the relationship between malnutrition diagnosed using the GLIM criteria and changes in nutritional status within the first 7 days of treatment in the NCU. Results are shown in Table 3. The interaction between the group and the time for RFMT and MAMC was significant. RFMT and MAMC were reduced to a greater extent in participants without malnutrition. (*β* = 1.85, 95% CI 0.45–3.24, *P* = 0.01;* β* = 2.09, 95% CI 0.03–4.16, *P* = 0.047, respectively). The increase of preperitoneal visceral is more pronounced in participants without malnutrition (*β* = −1.50, 95% CI − 2.91 to − 0.09, *P* = 0.037). The relationships between the GLIM criteria for malnutrition and changes in CC, and SFT during the first 7 days of the NCU stay were not statistically significant.

**Table 3:** Change of nutritional status in first 7 days.

Mean (95% CI)	Well-nourished	Malnourished	Group * time^a^		
	n = 35	n = 26	*β* (95% CI)	*Ward X*^*2*^	*P*
RFMT (mm)					
Day 1	15.46 (9.74 to 11.98)	10.56 (9.23 to 11.88)	1.85 (0.45 to 3.24)	6.701	0.010
Day 7	13.92 (12.75 to 15.09)	10.86 (9.74 to 11.98)			
Difference	− 1.54 (− 2.35 to − 0.74)	0.30 (− 0.84 to 1.45)			
CC (cm)					
Day 1	34.46 (33.23 to 35.69)	31.02 (29.61 to 32.42)	0.99 (− 0.16 to 2.15)	2.839	0.092
Day 7	33.56 (32.53 to 34.58)	31.11 (29.82 to 32.39)			
Difference	− 0.90 (− 1.59 to − 0.21)	0.09 (− 0.83 to 1.02)			
MAMC					
Day 1	25.23 (24.13 to 26.33)	22.34 (21.35 to 22.33)	2.09 (0.03 to 4.16)	3.935	0.047
Day 7	23.61 (21.82 to 25.40)	22.81 (21.57 to 24.05)			
Difference	− 1.62 (− 3.49 to 0.25)	0.47 (− 0.41 to 1.35)			
PVFT (mm)					
Day 1	13.27 (11.69 to 14.85)	9.71 (7.65 to 11.77)	− 1.50 (− 2.91 to − 0.09)	4.386	0.037
Day 7	15.02 (12.98 to 17.06)	9.96 (8.21 to 11.70)			
Difference	1.75 (0.52 to 2.98)	0.24 (− 0.45 to 0.94)			
SFT (mm)					
Day 1	12.80 (10.80 to 14.81)	10.88 (8.74 to 13.02)	1.09 (− 0.84 to 3.01)	1.220	0.269
Day 7	11.71 (10.07 to 13.38)	10.89 (8.67 to 13.11)			
Mean difference	− 1.08 (− 2.68 to 0.52)	0.01 (− 1.19 to 1.21)			

*RFMT* Rectus femoris muscle thickness, *CC* Calf circumference, *MAMC* Mid-arm muscle circumference, *PVFT* Preperitoneal visceral fat thickness, *SFT* subcutaneous fat thickness.

^a^Interactive effects of group and time, reference to well-nourished participants. Regression coefficients is calculated by using generalized estimating equations.

## Discussion

### Summary and interpretation

The results of our study show demonstrate the significant diagnostic efficacy of GLIM criteria in identifying malnutrition among neurocritically ill patients. We observed that the GLIM criteria possess a strong predictive capacity for adverse clinical outcomes during NCU hospitalisation and show a potential trend to predict the changes of rectus femoris muscle thickness, mid-arm muscle circumference and preperitoneal visceral fat thickness. These results suggest that malnutrition diagnosed using GLIM criteria had a positive validity on the prognosis of patients with neurocritical illness and their response to nutritional support.

The prevalence of malnutrition at NCU admission in our study was 28% according to the SGA criteria, which is lower than that reported in previous studies conducted in the ICU^30^. However, the prevalence of malnutrition according to the GLIM criteria in our study was relatively consistent with that in previous studies^16,31^. The main reason for this difference may be attributed to the utilization of ultrasound imaging for muscle assessment. The GLIM criteria cover sarcopenia and emphasize the use of any available modality for muscle mass assessment^25^. In our study, 49% of participants were diagnosed with malnutrition according to the GLIM criteria, and the highest positive rate of the GLIM criteria was reduced muscle mass (0.41). Overweight and obesity are widely recognised risk factors for stroke^32^, and most participants in our study had stroke as their primary diagnosis. Subjective assessment of malnutrition, especially muscle mass reduction, may be limited in overweight and obese patients^33^. In addition, muscle mass, rather than body weight or BMI, can accurately reflect the nutritional status of patients with critically ill^11^. Ultrasound imaging was used as an objective tool for muscle assessment in our study; it directly measured the thickness of the rectus femoris to eliminate bias due to subjective assessment. This may explain the higher prevalence rate according to the GLIM criteria and the higher sensitivity of these criteria.

Similar to previous studies, malnutrition as determined using the GLIM criteria was strongly associated with short-term adverse outcomes in critically ill patients. A study conducted in critically ill patients with COVID-19 showed that patients with GLIM-determined malnutrition have a longer ICU length of stay and about four times higher mortality than well-nourished patients^16^. In our study, malnutrition diagnosis based on the GLIM criteria caused approximately three to four adverse clinical outcomes when compared to patients without malnutrition. Adverse clinical outcomes in our study included clinical death, haemodynamic instability, and major complications in the NCU. Further analysis of our results showed that patients with severe malnutrition had a six times mortality rate than well-nourished patients; however, this result was not statistically significant after adjusting for age, sex, CCI score, and SOFA score. This result is consistent with a previous meta-analysis^14^, which pooled the evidence of the prognostic effect of the GLIM criteria in critically ill patients; evidence of the predictive value of the GLIM criteria in mortality is still lacking, and only two studies, different results were obtained^16,31^. This could be attributed to the relatively low short-term mortality among NCU patients, which is closely related to the disease status at admission^34^. Haemodynamic instability, defined as the use of dopamine or epinephrine during NCU hospitalisation, was a significant component of primary outcome in our study, and has been validated as a strong indicator of clinical death^35^. Previous study have suggested that malnutrition in critically ill patients can disrupt iron metabolism, diminish erythropoiesis, and shorten erythrocyte lifespan, consequently resulting in decreased erythrocyte count and impaired oxygen-carrying capacity, thereby predisposing patients to anemia^36^. Factors such as decreased blood viscosity and vasodilation due to anemia can lead to reduced cardiac afterload and alterations in hemodynamics^37^. Our results indicated that patients with GLIM-defined malnutrition have a 2.6 to 3.7 times rate of haemodynamic instability than well-nourished patients, and the presence of GLIM criteria malnutrition on NCU admission may be associated with a higher risk of death and worse disease status. In addition, the GLIM criteria-defined malnutrition showed a prognostic effect of NCU trans-out destinations; patients in good nutrition were more likely to return home, instead of staying in the hospital ward. This may also be part of the reason for the non-significant difference in the length of NCU stay between patients with and without malnutrition.

Aggressive nutritional support may not benefit certain critically ill patients^38^. Thus, one of the important purposes of identifying malnutrition is to distinguish individuals who would benefit the most from nutrition therapy. In our study, all participants received a relative high calorie delivery (> 20 kcal/kg/d), which could be viewed as implementing of aggressive nutritional support. Repeated measurements on 61 participants showed that, patients with malnutrition showed a trend to lose less muscle in thighs and upper limbs than that in well-nourished patients during the first seven days of NCU stay. Those results indicated that GLIM criteria could serve as an effective tool for differentiating individuals with varied responses to aggressive nutritional support, which partly consistent with a previous study conducted in adult medical inpatients^39^. However, another prospective study conducted in the ICU showed that patients with lower BMI had less rectus femoris cross-sectional areas loss in the first seven days, but not in patients with GLIM-defined malnutrition^31^, suggested that further study is needed to verify the predictive effect of GLIM criteria on the efficacy of nutritional support in critical care units. In addition, previous studies have described a phenomenon in which patients with critical illnesses have greater fat mass gain and muscle loss after ICU discharge^40–42^. Our results suggested that this phenomenon may commence upon admission to the critical care unit and is associated to the nutritional status of the patients. Specifically, well-nourished patients have a greater increase in visceral fat during the NCU stay. Increased visceral fat thickness is recognised as a risk factor for atherosclerosis^43^, demonstrates the potential adverse effects of aggressive nutritional support and underscoring the importance of personalised nutritional interventions tailored to patients' individual nutritional statuses. Nevertheless, it is noteworthy that the results of repeated measurements were derived from a relatively small simple size, potentially lacking sufficient test power to exclude the possibility of false positives. Further validation is required in future studies with larger sample sizes.

### Limitations

Our study had several limitations. The diagnosis of malnutrition using the SGA or GLIM criteria was conducted by the same dietitian, which may have introduced a potential bias. In our study, subjective assessment preceded objective assessment to limit bias. More than half of the participants missed the nutritional assessment within 48 h on day 7 because of NCU treatments or discharge, which may introduce potential bias to our exploratory results. However, the difference between the two groups was in terms of relative equality, which partly controlled for bias. The exploratory results in our study are derived from a relatively small simple size, that may not have enough power of statistic test. In addition, our study conducted a short-term follow-up during NCU hospitalisation, and long-term follow-up is still ongoing to assess the long-term effects of GLIM-defined malnutrition in patients with neurocritical illnesses.

### Implications

Our study provides preliminary evidence to validate the GLIM criteria for the diagnosis of malnutrition in patients with neurocritical illness. Indicates the importance of using available tools or equipment to screen for or assess malnutrition in patients with neurocritical illnesses. In addition, Ultrasound imaging as an objective assessment of muscle quality has potential value in the diagnosis of malnutrition as well as the monitoring of nutritional status, muscle and fat thickness measured by ultrasound imaging may be more sensitive for detecting changes in nutritional status than anthropometric measurements, such as mid-arm circumference or calf circumference. This demonstrates the potential value of ultrasound imaging as safe, reproducible, and easy-to-operate equipment for bedside nutritional assessment in critically ill patients.

## Conclusions

The GLIM criteria demonstrate a strong diagnostic efficacy for malnutrition, along with significant predictive validity for adverse clinical outcomes. Further studies are needed to validate the long-term effects of GLIM-defined malnutrition in patients with neurocritical illness and to extend the application of GLIM criteria to other critically ill patients.

### Supplementary Information

Supplementary Information.

## Data Availability

The datasets used and/or analysed during the current study are available from the corresponding author on reasonable request.
