# Supplementary material for: The predictive value of GLIM criteria on clinical outcomes and responses to nutritional support in patients with neurocritical illnesses
Source: Sci Rep. 2024 Jul 1;14:15061. doi: 10.1038/s41598-024-65994-2 (PMC11219829; doi:10.1038/s41598-024-65994-2)
Supplement: Supplementary file 1 — Supplementary Information. [file 41598_2024_65994_MOESM1_ESM.docx]

**Supplementary material to the manuscript**

Table S1. Baseline characteristics of participants.

Table S2. The diagnosis effect of each phenotypic and etiologic criteria.

Table S3. Average calorie intake in first 7 days.

Text S1: Nutritional support protocol.

Table S1. Baseline characteristics of participants.

|  | n=135 |
| --- | --- |
| Age (years), mean±SD | 62.10±13.34 |
| Female, n (%) | 50(37.0) |
| BMI, mean±SD | 24.33±4.80 |
| <18.5, n(%) | 14(10.4) |
| 18.5~24.9, n(%) | 64(47.4) |
| >24.9, n(%) | 57(42.2) |
| Primary diagnosis, n (%) |  |
| Stroke | 101(74.8) |
| Encephalitis and Meningitis | 16(11.9) |
| Status epilepticus | 10(7.4) |
| Acute nontraumatic weakness | 8(5.9) |
| SOFA, mean±SD | 5.20±2.06 |
| APACHE II, mean±SD | 14.36±4.93 |
| CCI, n (%) |  |
| 0~1 | 95(70.4) |
| ≥2 | 40(29.6) |
| GCS, median[IQR] | 9 [7,12] |
| Length of NCU stay (days), median [IQR] | 8[5,15] |
| NCU transfer out destination, n (%) |  |
| home | 32(23.7) |
| hospital ward | 91(67.4) |
| others | 12(8.9) |
| Nutritional assessment in day 7, n(%) | 61(45.2) |

Abbreviations: SD: Standard deviation; BMI: Body Mass Index; SOFA: Sequential Organ Failure Assessment; APACHE II: Acute Physiology and Chronic Health Evaluation II Chronic Health Evaluation II; CCI: Charlson Comorbidity Index; GCS: Glasgow Coma Scale; IQR: interquartile range; NCU: Neurocritical Care Unite.

Table S2. The diagnosis effect of each phenotypic and etiologic criteria.

|  | Positive rate | Sensi  tivity | Speci  ficity | PPV | NPV | PLR | NLR | Pre-cision | *kappa* (95% CI) |
| --- | --- | --- | --- | --- | --- | --- | --- | --- | --- |
| GLIM criteria | 0.49 | 0.95 | 0.69 | 0.55 | 0.97 | 3.06 | 0.07 | 0.76 | 0.52 (0.39—0.65) |
| Weight loss | 0.21 | 0.64 | 0.98 | 0.93 | 0.86 | 32.0 | 0.37 | 0.87 | 0.68 (0.54—0.82) |
| Low BMI | 0.16 | 0.38 | 0.95 | 0.76 | 0.77 | 7.60 | 0.65 | 0.77 | 0.38 (0.21—0.55) |
| Reduce muscle mass | 0.41 | 0.67 | 0.71 | 0.51 | 0.83 | 2.31 | 0.46 | 0.70 | 0.35 (0.19—0.51) |
| Reduce food intake/assimilation | 0.36 | 0.52 | 0.72 | 0.46 | 0.77 | 1.86 | 0.67 | 0.66 | 0.24 (0.06—0.41) |
| Inflammation | 0.99 | 1 | 0.02 | 0.32 | 1 | 1.02 | 0.00 | 0.87 | 0.01 (-0.01 - 0.03) |

Abbreviations: PPV: Positive Predictive Value; NPV: Negative Predictive Value; PLR: Positive Likelihood Ratio; NLR: Negative Likelihood Ratio; CI: Confidence Interval; BMI: Body Mass Index.

Table S3. Average calorie intake in first 7 days.

| Nutritional status | Average calorie intake (kcal) | | | | | | | *F* | *P* |
| --- | --- | --- | --- | --- | --- | --- | --- | --- | --- |
|  | Day 1 | Day 2 | Day 3 | Day 4 | Day 5 | Day 6 | Day 7 |  |  |
| Well-nutrition (n=69) | 789.57 | 1460.71 | 1567.29 | 1603.67 | 1603.27 | 1640.71 | 1651.71 | 1.688 | 0.199 |
| Malnutrition (n=66) | 704.04 | 1413.65 | 1522.89 | 1527.65 | 1547.89 | 1566.15 | 1611.92 |  |  |

Text S1: Nutritional support protocol

For patients with BMI <30, calorie intake target is settled as 20~25kcal/kg/d at beginning, rising to 25~30kcal/kg/d in 7 to 10 days. For patients with BMI between 30 and 50, support 11~14kcal/kg/d, and for patients with BMI >50, target is settled as 22~25kcal/kg (ideal body weight) per day. Ideal body weight are calculated based the flowing formula: [(height-150)×0.6+50] (kg). Nasogastric tube is the first choice for delivery. If there are significant complications, or if calorie goals cannot be met, nasoenteric tube or parenteral nutrition pathway would be added. 1.5kcal/ml calorie, non-element nutrient solution containing cellulose is the first choice, if adverse reactions or medical conditions require, consider using 0.9~1.5kcal/ml elements or a low-sugar diet, additional protein and trace elements are added if needed.
